# Quantifying replication stress in cancer without proliferation confounding

**DOI:** 10.15698/cst2025.10.312

**Published:** 2025-10-28

**Authors:** Philipp Jungk, Maik Kschischo

**Affiliations:** 1 Institute for Computer Science, University of Koblenz, Koblenz, Rhineland-Palatinate, Germany.

**Keywords:** replication stress, gene expression signatures, cell proliferation, chromosomal instability, non-homologous end-joining, mismatch repair

## Abstract

Replication stress (RS) is a major driver of genomic instability and cancer development through impaired DNA replication that can lead to chromosomal instability (CIN). Although RS is mechanistically linked to CIN, its relationship with cellular proliferation is complex. Depending on the context, RS can either promote or suppress cell growth. Existing RS gene expression signatures overlook this complexity, relying on the overexpression of oncogenes such as *MYC*, which introduces a proliferation bias. To disentangle genuine RS from confounding cell cycle and proliferation transcriptional profiles, we developed and validated a novel gene expression signature that accurately predicts RS independently of oncogene activity. This tumorigenic RS signature (TRSS) captures RS-related transcriptional changes across diverse cellular contexts, enabling a more robust and proliferation-independent measure of RS in both experimental and clinical samples. Applying our signature to patient data, we discovered a link between RS and the non-homologous end-joining (NHEJ) DNA repair pathway. Specifically, we observed that *MSH2* and *MSH6* - core components of mismatch repair - are associated with elevated RS and may indicate a shift toward NHEJ-mediated repair under stress conditions. Our study provides a refined approach to quantify RS and sheds light on its broader impact on DNA repair network dynamics.

## Abbreviations

APH - aphidicolin,

ARSS - Acute Replication Stress Signature,

CIN - chromosomal instability,

CPTAC - Clinical Proteomic Tumor Analysis Consortium,

DSB - double-strand break,

GSEA - gene set enrichment analysis,

NHEJ - non-homologous end-joining,

RS - replication stress,

RSRD - RS response defects,

S-CIN - structural CIN,

SCS - structural complexity score,

TCGA - The Cancer Genome Atlas,

TRSS - Tumorigenic RS Signature.

## INTRODUCTION

Replication stress (RS) is a significant contributor to genomic instability and cancer development. It is defined as slowed or stalled DNA replication caused by factors like unusual DNA structures, impaired origin licensing, nucleotide pool depletion and transcription-replication collisions 1. These disruptions stall or collapse replication forks, leading to under-replicated DNA, double-strand breaks (DSB) and ultimately to structural chromosomal instability (S-CIN). RS also links S-CIN and whole chromosomal instability (W-CIN), describing the continuous gain or loss of entire chromosomes 2, through mitotic errors caused by mechanisms like impaired kinetochore attachment and abnormal spindle assembly 3,4,5. Chromosomal instability (CIN), a hallmark of cancers, is thought to promote tumor growth 6 by deregulating pathways via gains in oncogenes (e.g. *CCNE1*, *MYC*) or losses in tumor suppressors (e.g. *PTEN*, *RB1*) 7,8.

Accurately quantifying RS based on omics-data is essential for linking it to other tumor-related processes, translating findings from biological experiments into primary tumor databases. In cell line experiments RS is commonly induced by drug treatment (e.g. aphidicolin (APH) or hydroxyurea), oncogene overexpression (e.g. *MYC*, or *CCNE1*), or gene knockouts (e.g. *DHX9*) 9,10,11. While effective in controlled laboratory settings, each method also alters cellular programs unrelated to RS, skewing phenotypic correlations if examined in isolation. For instance, oncogene overexpression may bias the transcriptome toward genes linked to premature cell cycle progression, potentially obscuring RS-specific effects like the activation of DSB repair pathways in S-phase 1,12,13,14. Despite these limitations, current gene expression-based RS quantifications emphasize oncogene-related phenotypes 15,16, likely due to their established association to RS in cell lines and frequent natural occurrence in primary tumors 10.

CIN was suggested to paradoxically affect proliferation, enhancing fitness in some context while impairing it in others 6,17. Further studies have extended upon these discussions, including evidence of a similar paradoxical relationship between RS and proliferation. For instance, CIN signatures were shown to positively correlate with the proliferation markers like *PCNA* and *MKI67*, as well as proliferation scores in primary tumors, suggesting that genomic instability might drive tumor growth in advanced stages of cancers 17,18,19. CIN, assessed through comparative genomic hybridization and image cytometry, has been linked to gene expressions indicating cancer development and cellular growth in breast cancers 20. In mouse models and murine embryonic fibroblasts, *BubR1^+/-^Apc^Min/+^* induced CIN led to the development of higher growth rates 21. Similarly, a causal link to proliferation is suggested by nucleotide depletion in highly proliferative cells inducing RS 22,23. However, evidence for this positive association in cell lines is limited. Experiments suggesting that genomic instability increases proliferation required additional defects in cell cycle regulators (e.g. *ATR*, *ATM*), alongside oncogene-induced RS 24,25. Inhibiting the DNA damage checkpoint assuring faithfully replicated DNA in oncogene-driven proliferation environments may increase proliferation and overshadowing CIN/RS-dependent effects. Further experiments in cell lines do suggest that CIN and RS generally reduce cellular fitness. For example, even low RS levels induced by 100 nM APH were shown to decrease proliferation in cell lines 26, while increased karyotype heterogeneity in NCI60 cells correlates with slower doubling times 17. Gene set enrichment analysis (GSEA) of CIN-associated genes in breast cancer cell lines indicated reduced cell cycle activity and a shift of their transcriptome toward a mesenchymal state 17,27. Interestingly, some mouse models harboring CIN were shown to reduce proliferation. For instance, *Pten^+/-^Bub1^-/H^* mice exhibited increased abnormal chromosomal numbers, while *PCNA*-positive cells were reduced compared to *Pten^+/-^* mice with stable chromosome numbers 28. Another study found a simultaneous decrease in expression of the proliferation marker *Ki67* and an increase in levels of DNA damage as well as aneuploidy in *SA1*-deficient mice 29. While most studies on the association of genomic instability and proliferation focus on CIN, the limited evidence on RS displaying similar trends, suggests that the CIN-proliferation relationship extends to RS 2,30,31,32.

To determine a suitable quantification of RS, we first systematically evaluate existing RS scores and analyze their association with a chromosomal complexity score while accounting for the influence of other cancer hallmarks. Using public gene expression data from various RS-inducing methods, we develop a new RS signature that performs well even on unseen experimental data, while minimizing the confounding effects of oncogene overexpression and capturing the complex relationship between proliferation to RS. Finally, we apply this novel RS signature to large genomic databases to uncover a proteomic link to the non-homologous end-joining pathway, demonstrating its utility for uncovering new insights into cancer biology.

## RESULTS

### Existing signatures fail to predict RS in cell lines

To estimate the performance of previously published RS signatures, we gathered public RNA-sequencing data from various studies using different methods for RS induction (**Table 1**): Three of the methods used RS-inducing oncogene overexpression (*CCNE1*, *CDC25A*, *MYC*) 33,34, while three additional methods induced RS by silencing genes affecting RS-pathways (*DHX9*, *CDA*, *SMAD4*) 11,35. Samples were divided into those with and without RS.

**Table 1:** . Samples with gene amplification or knockout to induce replication stress.

***Study_ID***	***Cell line***	***RS Technique***	***GEO series***
*GSE171845*	RPE_1	CCNE1	GSE171845
*GSE185512*	RPE_1	CDC25A, CCNE1, MYC	GSE185512
*GSE185512*	RPE_1_NOp53	CDC25A, CCNE1, MYC	GSE185512
*GSE185512*	BT549	CDC25A, CCNE1, MYC	GSE185512
*GSE185512*	MDA_MB231	CDC25A, CCNE1, MYC	GSE185512
*GSE185512*	HCC1806	CDC25A, CCNE1, MYC	GSE185512
*GSE244103*	H446	DHX9_KO	GSE244103
*GSE244103*	H82	DHX9_KO	GSE244103
*GSE244103*	H196	DHX9_KO	GSE244103
*GSE252544*	Mia_PACA_2	CDA_KO	GSE252544
*GSE292334*	HCT116	SMAD4_KO	GSE292334
*GSE292334*	HT29	SMAD4_KO	GSE292334

**Figure 1:**
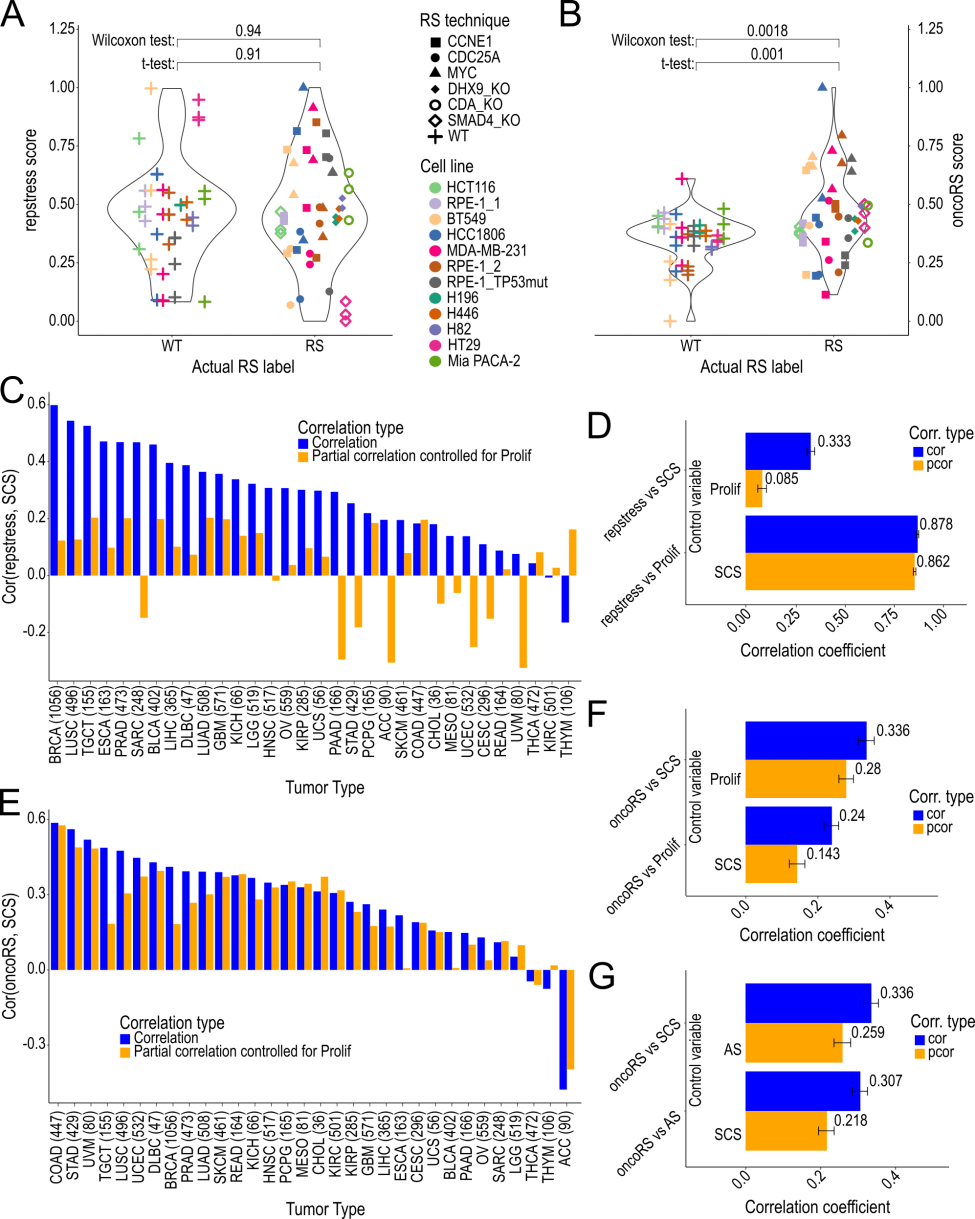
FIGURE 1: Assessment of RS prediction scores and their robustness across multiple studies and associated phenotype scores. Evaluation of two replication stress scores on a dataset including six different ways of inducing replication stress in multiple cell lines. The y-axis represents the replication stress levels as predicted by the repstress score **(A)** and the oncoRS score **(B)**, while the x-axis distinguishes between the actual status of samples either with replication stress (RS) or without (WT). **(C)** Spearman correlation of the repstress score and the SCS within tumors of TCGA. The y-axis visualizes the non-adjusted (blue) correlation coefficient and the partial (orange) correlation coefficient between repstress score and SCS accounting for proliferation in each tumor type (x-axis). **(D)** Pan-cancer correlation between repstress score and SCS and proliferation with (orange) and without (blue) correction for confounding effects of the annotated control variable using partial correlation. The y-axis shows the comparison pair, while the x-axis represents the (partial) correlation coefficient, which is additionally written next to the bar. **(E)** Spearman correlation of the oncoRS score and the SCS within tumors of TCGA. The y-axis visualizes the non-adjusted (blue) correlation coefficient and the partial (orange) correlation coefficient between oncoRS score and SCS accounting for proliferation in each tumor type (x-axis). **(F, G)** Pan-cancer correlation between oncoRS score and SCS, proliferation **(F)** and aneuploidy **(G)** with (orange) and without (blue) correction for confounding effects of the annotated control variable using partial correlation. The y-axis shows the comparison pair, while the x-axis represents the (partial) correlation coefficient, which is additionally written next to the bar. Error bars in **(D, F, G)** represent the 95% confidence interval. SCS: Structural Complexity Score; TCGA: The Cancer Genome Atlas; AS: Aneuploidy Score; Prolif: proliferation.

We applied two published RS signatures to estimate the RS in the dataset’s samples: (i) The *repstress* score based on *MYC* overexpression, DNA damage checkpoint activation and sensitivity of checkpoint inhibitors and (ii) the *oncoRS* score based on oncogene overexpression in cell lines and oncogene amplification in primary tumors 15,16. Notably, samples with and without RS-induction displayed no difference in the repstress scores, indicating that the repstress score was not able to predict RS status (**Figure 1A**), while the oncoRS signature was only able to differentiate RS from non-RS in samples with *MYC*-based RS-induction (**Figure 1B**).

To further assess the physiological reliability of these signatures, we used the structural complexity score (SCS), which quantifies the heterogeneity of a sample’s copy number landscape to approximate S-CIN 2. Further, we used a gene expression-based quantification of the proliferation rate 36 and an aneuploidy score based on the absolute number of arm-level alterations of a sample 37.

We evaluated the association between RS scores and SCS in primary tumors of The Cancer Genome Atlas (TCGA) by calculating the Spearman correlation. Using partial correlation, we investigated the confounding of this RS-SCS association by proliferation, potentially resulting from bias toward oncogene and cell cycle activities.

The repstress score displayed a strong correlation with proliferation (Supplementary Figure S1) and a significant association with the SCS within (**Figure 1C**) and across (**Figure 1D**) TCGA tumor types. However, this association with the SCS was reduced significantly when controlling for proliferation. Additionally, more than half of the repstress signature genes are cell cycle genes (9/17, 53%) and most signature genes display a strong correlation to the proliferation score and gene expressions of the proliferation markers *PCNA* and *MKI67* (Supplementary Table S1). In combination this suggests that the repstress score-SCS association is largely mediated by proliferation.

The correlation of the oncoRS score with the SCS remained largely unaffected after accounting for proliferation (**Figure 1E, F**). Since the oncoRS score is partially based on oncogene amplification, we further investigated the signature for confounding by arm-level aneuploidies. A pan-cancer comparison of oncoRS and aneuploidy revealed a similar correlation as with the SCS. Partial correlation showed that the correlation of oncoRS and the S-CIN measure may be influenced by aneuploidies, as it resulted in a mild reduction in the oncoRS-SCS association (**Figure 1G**). Independent validation of the oncoRS signature genes remains limited with *NAT10* being the only gene confirmed to display an association with RS markers in tumors 16.

### Moderate APH dosage leads to a non-physiological RS response with an accumulation of cells in S-phase

**Figure 2:**
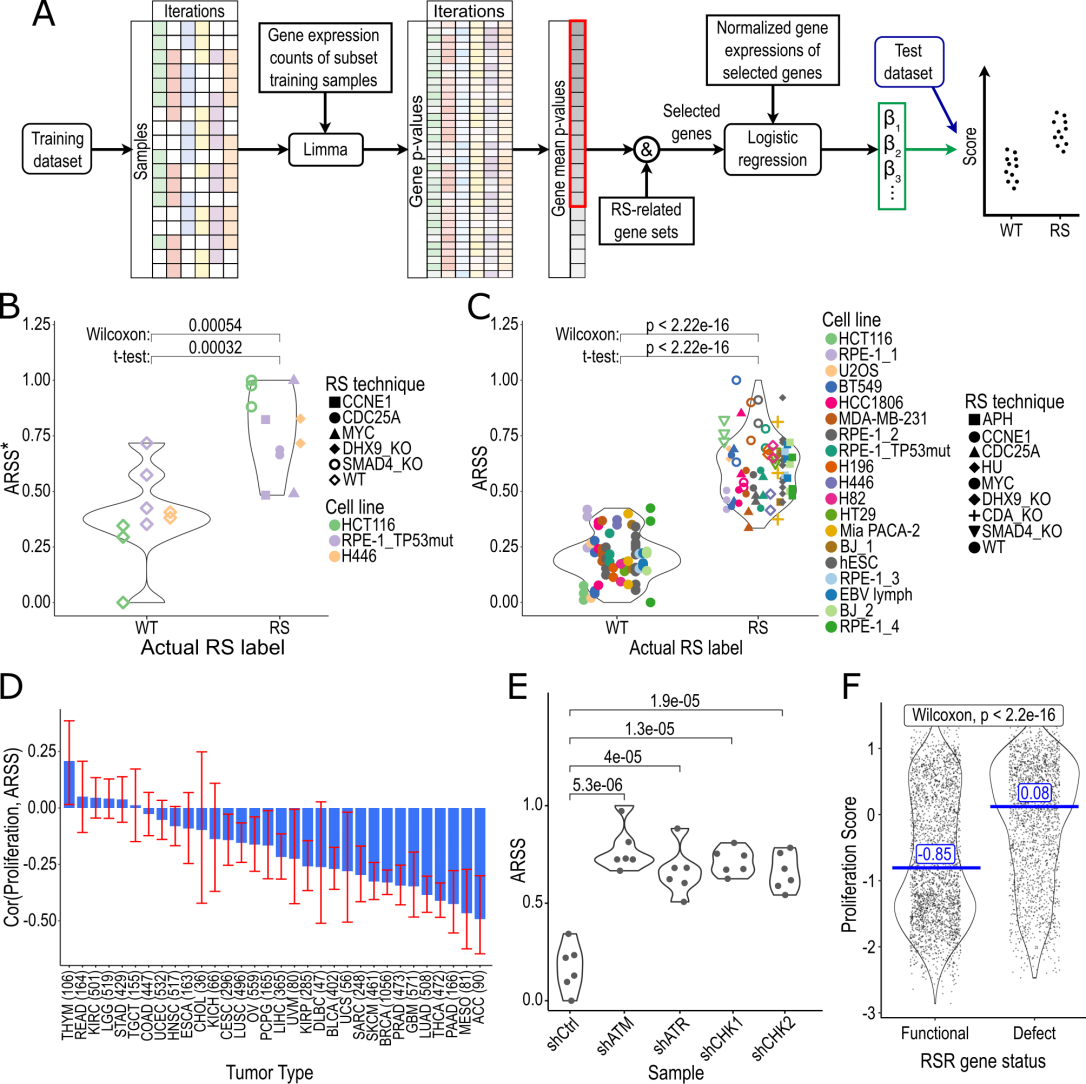
FIGURE 2: Development and interpretation of a signature of acute replication stress. **(A)** Workflow of the RS prediction model development. Estimating consistently differentially expressed genes using a permutation approach, limma-based differential gene expression determination and calculating gene weights using regularized logistic regression. **(B)** Comparison of the RS scores predicted by a signature for acute RS (ARSS*) between samples with (RS) and without (WT) replication stress on an unseen test data set. **(C)** Predictions of the ARSS on the entire dataset combining eleven studies and eight RS induction methods compared to the actual RS status grouped into samples with (RS) and without (WT) replication stress. **(D)** Spearman correlation between the ARSS and a proliferative score (y-axis) in different TCGA tumor types (x-axis). **(E)** Acute RS score in samples with functional RS response (shCtrl) and with silenced gene expression of RS response genes (shATM, shATR, shCHK1, shCHK2). **(F)** Comparison of proliferation between TCGA samples with (Defect) and without (Functional) mutations or copy number losses in RS response genes (ATR, ATM, CHEK1, CHEK2). RS: Replication stress; ARSS: Acute replication stress signature; TCGA: The Cancer Genome Atlas; RSR: Replication stress response.

We aimed to derive a RS signature without intrinsic biases toward proliferation, copy number alterations (CNA), or oncogene-related processes. To achieve this, we expanded the dataset in **Table 1** to include drug-induced RS using APH and hydroxyurea (Supplementary Table S2). In our systematic approach (**Figure 2A**), we first calculated differentially expressed genes in RS samples using a bootstrap approach of the limma-voom pipeline 38,39. To further reduce the dimensionality of the selection, we integrated prior biological knowledge by including only genes involved in any processes potentially associated with RS, such as DNA replication and repair among others (Supplementary Table S3). The top-ranked genes were then selected for the signature. In the last step, batch corrected log-expressions of the signature genes were used to train a regularized logistic regression model.

To assess the modeling approach and selected hyperparameters, we applied this signature ( referred to as Acute Replication Stress Signature (ARSS*) ( to a set of unseen test data. ARSS* scores were computed by a linear combination of the model coefficients and the expression values of the signature genes, which resulted in a reliable prediction of RS status (**Figure 2B**).

After testing the performance on unseen data, we retrained the model on the full data set to leverage all available transcriptomic data for downstream analysis. We refer to this final model including all the updated coefficients as ARSS (**Table 2**) and used it for further analysis. The distribution of the ARSS across all samples (including training samples) is shown in **Figure 2C**.

**Table 2:** Genes in Acute Replication Stress Signature (ARSS) including their weights.

	**Gene**	**coef**
1	TFDP2	2.596
2	SF3A3	2.518
3	BTG2	2.013
4	RADX	1.063
5	BRME1	1.048
6	POLH	0.930
7	SUPT7L	0.575
8	RRM2B	0.534
9	CEP164	0.424
10	SUN2	0.259
11	REV3L	0.254
12	AOC2	0.193
13	PLK1	-0.039
14	PPP2R1A	-0.097
15	ERCC1	-0.097
16	VCL	-0.126
17	MCM6	-0.372
18	HINFP	-0.421
19	H2BC14	-0.728
20	NUCKS1	-0.861
21	POLR1D	-1.527
22	ERCC2	-1.924
23	FBXL7	-2.338
24	SPIDR	-2.440
25	PIF1	-2.577
26	H2AC14	-2.885
27	AKT3	-3.735
28	PPP2R5A	-4.588

Previously, using moderate APH dosages over 100 nM to induce RS has been criticized for halting cell cycle progression through checkpoint activation rather than a physiological response to RS 26. To assess whether the ARSS, which uses training samples with APH levels of 200-400 nM, is physiologically feasible in primary tumors, we compared its association to proliferation. As displayed in **Figure 2D**, ARSS is negatively correlated with proliferation in TCGA tumor types, contrasting the previous observation of increased proliferation in tumors with RS and genomic instability 20. To investigate the meaning of the ARSS, we used a dataset with defects in the RS response genes *CHK1/2*, *ATR*, *ATM* 24. Notably, samples with RS response defects (RSRD) displayed significantly higher levels of ARSS (**Figure 2E**). Defects in checkpoint genes are associated with an increase in proliferation in TCGA primary tumors, as indicated by higher proliferation rates in samples with heterozygous RSRD (**Figure 2F**), and in cell line experiments with silenced RS-response genes 24,25. However, disruption of the cell cycle by hydroxyurea was reported to expose an inability to recover from stalled replication forks 24. This suggests a high level of stress in these RSRD samples that might resemble samples with RS induced by moderate APH dosages as used in ARSS generation.

We performed Gene Set Enrichment Analysis 40 to identify pathways associated with both the ARSS scores and RSRD status. Samples were classified as RSRD if they contained a mutation or copy number loss in one of the RS response genes listed above. Common enrichment was observed in the *TP53* pathway (q < 0.01 for both signatures) and the gene set for downregulation of the *KRAS* signaling pathway (q < 0.03 for both signatures) 41. Both describe the inhibition of cell cycle progression at the G1/S checkpoint 42,43, which aligns with the observation of inhibited cell cycle progression in samples treated with moderate APH dosages 26. These results suggest that the samples with 200-400 nM APH treatment drive the ARSS to reflect conditions of severe RS leading to cell cycle arrest instead of tumorigenic RS.

### A novel RS signature highlights elevated proliferative cell cycle activity in primary tumors versus cell lines

**Figure 3:**
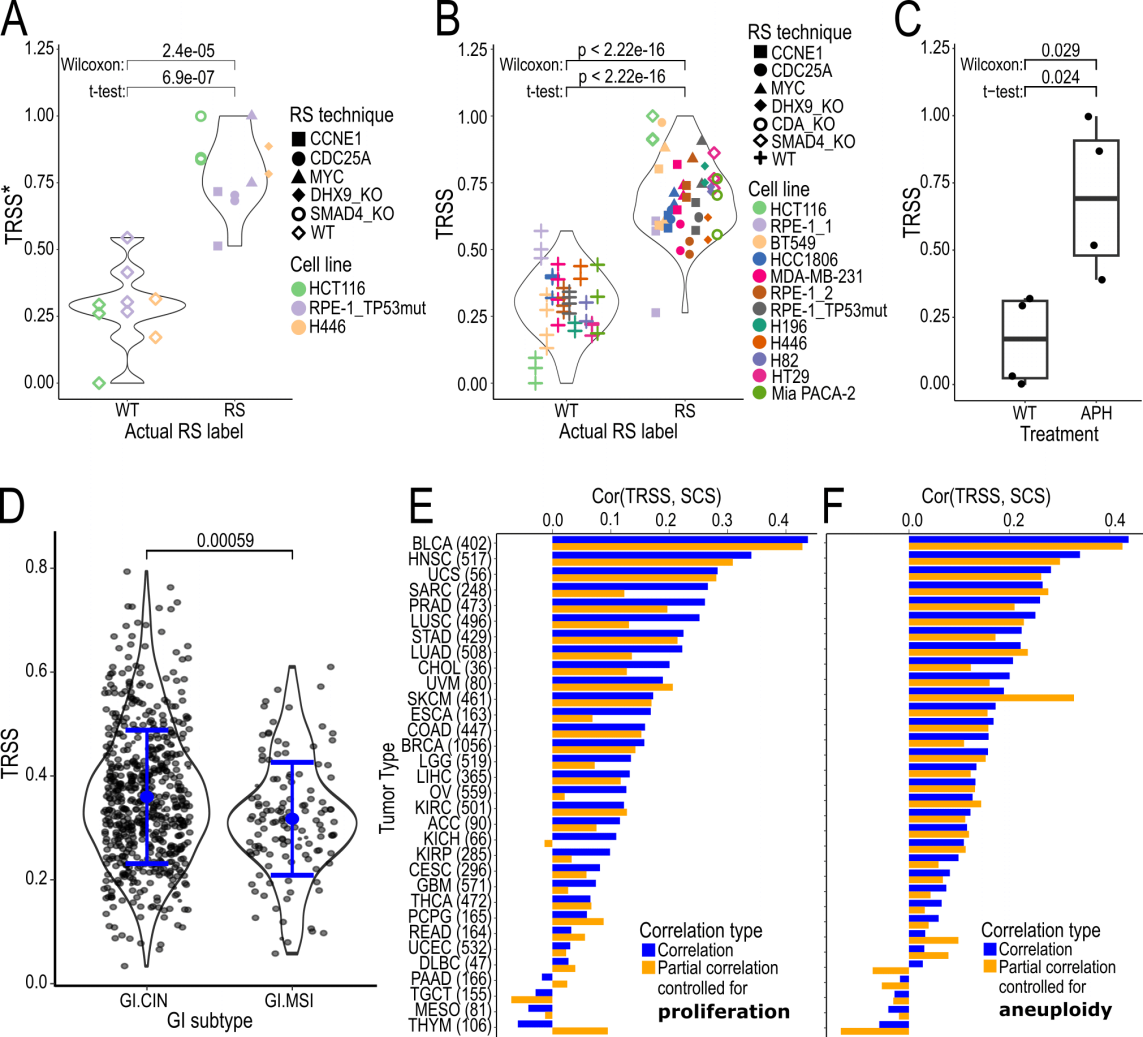
FIGURE 3: Validation and application of a novel RS signature for tumorigenic RS not confounded by proliferation and aneuploidy. ** (A)** Comparison of the RS scores predicted by a signature for tumorigenic RS (TRSS*) between samples with (RS) and without (WT) replication stress on an unseen test data set. **(B)** Predictions of the TRSS on the entire dataset combining eleven studies and eight RS induction methods compared to the actual RS status grouped into samples with (RS) and without (WT) replication stress. **(C)** Comparison of TRSS between aphidicolin-treated (200 nM; APH) and untreated (WT) samples. **(D)** TRSS distribution across CIN and MSI molecular subtypes in TCGA gastrointestinal cancers. **(E, F)** Spearman correlation of the TRSS and the SCS within tumors of TCGA. The y-axis visualizes the non-adjusted (blue) correlation coefficient and the partial (orange) correlation coefficient between repstress score and SCS accounting for proliferation **(E)** and aneuploidy **(F)** in each tumor type (x-axis). RS: Replication Stress; TRSS: Tumorigenic replication stress signature; TCGA: The Cancer Genome Atlas SCS: Structural Complexity Score.

To capture physiological, tumorigenic RS, we developed a second signature by excluding samples subjected to severe RS conditions, such as those treated with high concentrations of APH and hydroxyurea from the dataset (**Table 1**). This signature was trained using the same methodology (**Figure 2A**) on the reduced dataset, resulting in the Tumorigenic RS Signature (TRSS*). Applying the TRSS* to a held-out test set confirmed that the methodology was still able to differentiate RS and non-RS samples (**Figure 3A**). The final model was re-trained on the full dataset to obtain the final coefficients (**Table 3**). We will refer to this re-trained model as the TRSS. For comparison with **Figure 3A**, the distribution of the TRSS across all samples (including training samples) is shown in **Figure 3B**.

**Table 3:** Genes in Tumorigenic Replication Stress Signature (TRSS) including their weights.

	**Gene**	**coef**
1	ARID2	3.252
2	H4C8	2.956
3	TOP3A	2.630
4	TAF12	2.358
5	AOC2	2.063
6	BTG2	1.906
7	TFDP2	1.848
8	PHF8	1.729
9	ATF2	1.591
10	H2BC12	1.415
11	H2AC6	0.235
12	GLI1	-0.085
13	POLD4	-0.144
14	TUBB	-0.390
15	BCAM	-0.793
16	H4C14	-1.026
17	UFL1	-1.210
18	CMPK2	-1.329
19	FBXL7	-1.626
20	TP53	-2.693
21	PPP2R5A	-3.138

Next, we applied the TRSS to the aphidicolin-treated samples with the lowest dosage available in our dataset (**Table 1**). The TRSS retained its predictive capacity at the dosage of 200 nM APH (**Figure 3C**), while the two existing scores were again not able to predict treatment status (Supplementary Figure S1C and D). Further we found that molecular subtypes in gastrointestinal cancers (COAD, ESCA, READ, STAD) and UCEC tumors in the TCGA database associated with CIN or increased copy number alterations tend to display a higher TRSS than MSI or low CNA subtypes (**Figure 3D**; Supplementary Figure S2A).

To validate the biological relevance of this second RS signature, we examined its association to the SCS in TCGA tumors, while also testing whether this association was dependent on proliferation or aneuploidy using partial correlation. While proliferation had a minor effect on the association (**Figure 3E**), the confounding by aneuploidy was negligible (**Figure 3F**). Moreover, we found that the TRSS generally exhibited a weak correlation with proliferation (cor: 0.1) and aneuploidy (cor: 0.07), suggesting the TRSS captures an aspect of genomic instability independent of proliferative capacity or extensive, stable chromosomal changes.

In an additional analysis we included MSigDB hallmark gene set activities as covariates when modeling the association of TRSS and SCS. No single pathway accounted for a major portion of the association (Supplementary Figure S2B; Supplementary Methods).

**Figure 4:**
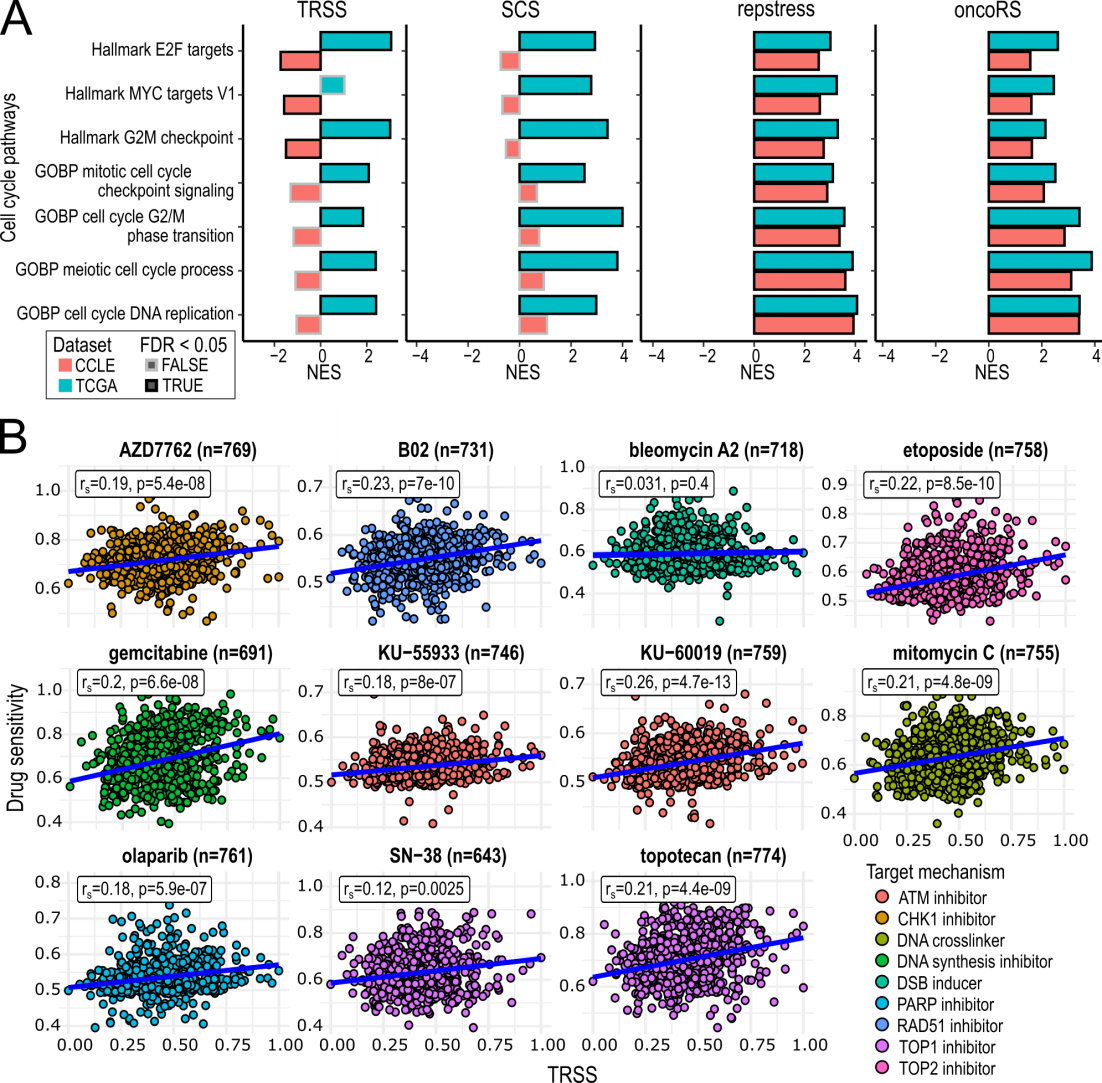
FIGURE 4: TRSS reflects context-dependent proliferative activity and predicts sensitivity to agents targeting faithful DNA replication. **(A)** Normalized enrichment score (NES) for gene sets representing distinct stages of the cell cycle shown in relation to the TRSS, and SCS, as well as the oncoRS, and repstress scores across TCGA (blue) and CCLE (red). Enrichment scores were calculated using gene set enrichment analysis. **(B)** Faceted scatterplots displaying the association of TRSS and drug sensitivity of eleven compounds available in the CTRP targeting DNA repair pathways or inducing DNA damage. RS: Replication Stress; TRSS: Tumorigenic replication stress signature; TCGA: The Cancer Genome Atlas; SCS: Structural Complexity Score; CCLE: Cancer cell line encyclopedia; NES: Normalized enrichment score; CTRP: Cancer Therapeutics Response Portal.

Next, we investigated whether the association between TRSS and proliferation might vary depending on biological context by examining differences in cell cycle activity between primary tumors and cell lines. Using GSEA, we calculated the enrichment of cell cycle-related gene sets from the gene ontology biological process 44,45 and hallmark collections 41 with respect to TRSS and SCS (**Figure 4A**; Supplementary Table S4). Interestingly, for both scores we found a significant upregulation of various cell cycle gene sets in TCGA primary tumors, whereas this upregulation was not observable in cell lines from the Cancer Cell Line Encyclopedia (CCLE). On the contrary, oncoRS and repstress scores displayed the same association with these cell cycle gene sets in cell lines and primary tumors. We asked if this enrichment implied a cell cycle-related increase of proliferation by examining the deregulation of leading-edge genes associated with the TRSS with a proliferation score and *PCNA* and *MKI67*. Indeed, in TCGA, leading-edge genes of the enriched pathways displayed a higher correlation with the three proliferation measures than non-leading-edge genes as determined by a two-sample Wilcoxon test (all p < 2.2e-16; Supplementary Figure S3A).

In cell lines on the other hand, the leading-edge genes indicated a reduction in cell cycle progression (all p < 2.2e-16; Supplementary Figure S3B). Thus, the TRSS captures the context-dependent association to proliferation, indicating an increase in cell cycle activity promoting proliferation in primary tumors, but not in cell lines.

### Prognostic and therapeutic associations of the TRSS

To assess the clinical relevance of the TRSS we first examined its association with overall survival across TCGA tumors. Notably, we did not observe a significant difference in survival between patients in the lowest (Q1) and highest (Q4) TRSS quartiles (Supplementary Figure S3C), suggesting that the TRSS does not independently stratify patient prognosis in this pan-cancer cohort.

To evaluate whether the TRSS reflects sensitivity to agents that interfere with faithful DNA replication, we selected compounds known to target vulnerabilities induced by RS 46,47 and analyzed the TRSS-associated drug sensitivity using data from the Cancer Therapeutics Response Portal (CTRP) 48,49,50. The selected drugs included inhibitors targeting key components of the DNA damage response such as ATM inhibitors (KU-55933 and KU-60019), a CHK1 inhibitor (AZD7762) and a PARP inhibitor (olaparib), as well as compounds inducing DNA damage, including topoisomerase inhibitors (topotecan, etoposide), and a DNA crosslinker (mitomycin C).

We observed a modest but consistent increase in sensitivity to these agents in samples with higher TRSS levels (**Figure 4B**). An analysis of point mutations and copy number alterations further revealed that the TRSS is associated with alterations in genes involved in DNA repair, replication fork protection, and the TP53 pathway (Supplemental Material).

These observations show that TRSS levels correspond to DNA repair pathway alterations and a sensitivity to RS-targeting compounds.

### Tumorigenic RS signature reveals MutSα-mediated regulation of double-stranded DNA recombination in proteomics data

**Figure 5:**
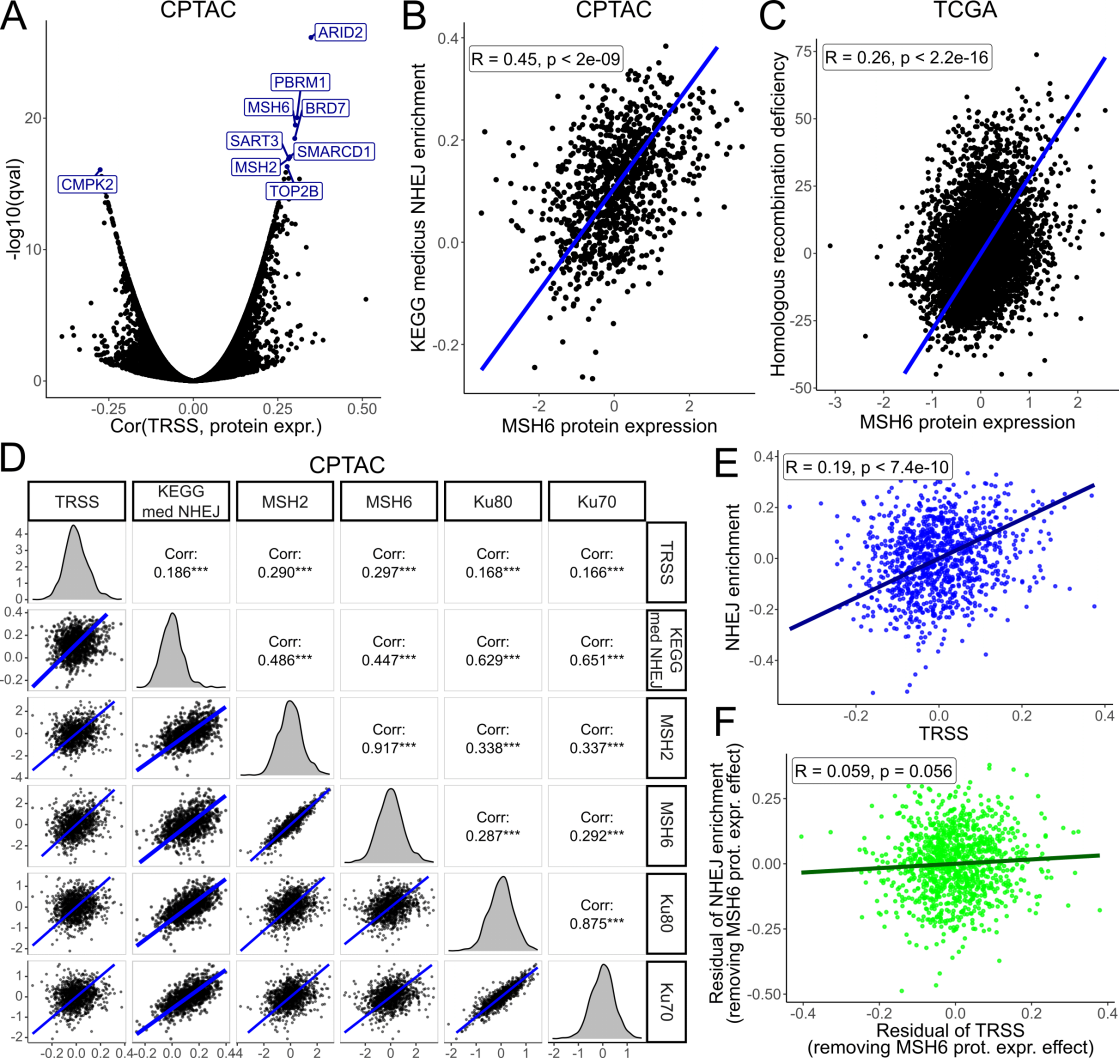
FIGURE 5: MSH6 protein expression is positively associated with the TRSS and NHEJ, potentially leading to low fidelity DNA repair and genomic scars resembling homologous recombination deficiency. **(A)** Volcano plot visualizing the spearman correlation and q-values of CPTAC protein expressions to the TRSS. **(B)** Activity in the KEGG medicus NHEJ gene set compared to the protein expression of MSH6 in CPTAC. **(C)** Amount of genomic scars typical for homologous recombination deficiencies compared to the protein expression of MSH6 in CPTAC. **(D)** A comparison of TRSS and NHEJ enrichment to protein expression of the proteins in the MSH2/6 complex and the Ku heterodimer in CPTAC. **(E)** Activity in the KEGG medicus NHEJ gene set compared to the TRSS in CPTAC. **(F)** Activity in the KEGG medicus NHEJ gene set compared to the TRSS after adjusting for the effects of MSH6 expression in CPTAC. TRSS: Tumorigenic replication stress signature; CPTAC: Clinical Proteomic Tumor Analysis Consortium; NHEJ: Non-homologous end-joining; TCGA: The Cancer Genome Atlas; KEGG: Kyoto Encyclopedia of Genes and Genomes

A recent study in yeast suggests that cellular stress can alter mRNA translation patterns 51. Using protein expression data from the Clinical Proteomic Tumor Analysis Consortium (CPTAC) database, we investigated proteomic effects of RS. Apart from ARID2, which is part of the TRSS signature (**Table 3**), two of the proteins most significantly correlated with the TRSS were *MSH6* and *MSH2*, which form the DNA mismatch repair complex MutSα (**Figure 5A**). *MSH6* protein expression and TRSS exhibited a Spearman correlation of 0.3, while *MSH2* protein expression displayed a correlation of 0.28 (q-value < 2e-14). Interestingly, no other MMR proteins demonstrated a comparably strong association with the TRSS (Supplementary Table S5). The two MutSα proteins were also among the top-ranking proteins in the TCGA proteomics dataset (both q-values < 2e-14), although it only includes about 300 (phospho-)proteins (Supplementary Figure S4A).

While sensing mismatches is the primary function of MutSα, its components are also implicated in the DSB repair pathways of non-homologues end-joining (NHEJ) and homologous recombination 52,53,54. Correlation analyses revealed that *MSH2/6* protein expression was significantly associated with single sample GSEA enrichment scores 55 in the KEGG medicus reference NHEJ gene set 56 (**Figure 5B**, Supplementary Figure S4B). *MSH2/6* also correlated with a score quantifying genomic scars resulting from homologous recombination deficiency 57 in TCGA, indicating low fidelity DSB repair (**Figure 5C**, Supplementary Figure S4C). Further analysis in CPTAC demonstrated that MutSα components as well as the TRSS were associated with Ku70 and Ku80 (**Figure 5D**), crucial components of the double-strand break repair via NHEJ 58. The relationship of MutSα and NHEJ also translated into an association between TRSS and NHEJ in CPTAC (**Figure 5E**, Supplementary Figure S4D). Interestingly, upon partial correlation to control for *MSH2/6* protein expression the association between TRSS and NHEJ activity was removed, suggesting that the association might be mediated by MutSα (**Figure 5F**, Supplementary Figure S4E).

## DISCUSSION

This study sought to evaluate the proficiencies and limitations of existing RS gene expression signatures and improve upon these limitations to elucidate new insights into the role of RS in tumorigenesis.

Using a dataset of labeled samples from the gene expression omnibus, we found that both the repstress score as well as the oncoRS score fail to reliably differentiate RS samples. The repstress score demonstrated an exaggerated dependence on genes associated with cell cycle progression. This is attributable to the selection of *MYC*-associated genes from the G2M-checkpoint and E2F target hallmark pathways, as supported by the strong association to a proliferation score, which also decouples the expected association between the repstress score and a measure for structural aneuploidies. While there are contexts in which proliferation drives RS, for example by causing nucleotide deficiency 22,23, RS can also induce cell cycle arrest, reducing proliferation 59. Moreover, recent studies suggest that RS may also be reduced as a result of adaptation to aneuploidy, which is accompanied by an increase in proliferation 60,61.

The oncoRS score is only targeted at identifying genes associated with oncogene-induced RS, as acknowledged by the original authors, likely limiting it from generalizing to RS originating from other sources. The signature may instead over-emphasize other oncogene-specific processes like cell cycle regulation as essential causes of RS 1,12,13,14. Methodologically, the score lacks validation of the entire signature, as only one signature gene is explored further. While the present study establishes a link between the oncoRS score and the SCS, both the signature and the SCS rely on copy number alterations in TCGA tumors. The reduced association when controlling for arm-level aneuploidies might indicate that the signature captures aneuploidy-related gene expression, rather than directly measuring RS related to CIN.

Our first alternative approach leveraged oncogene, gene knockout and drug-based induction of RS to detect transcriptional changes and define the ARSS. However, our analysis suggests that the samples with high APH concentrations might contain non-physiological levels of RS, which manifest itself through increased levels of cell cycle arrest as evidenced by the lower proliferation rate in samples with high ARSS. While a slight increase in checkpoint activation might be expected upon DNA damage resulting from RS, the APH samples might represent a gene expression profile of a checkpoint response to excessive DNA RS and DNA damage 26. The good predictive ability on samples with defective RS response indicates that RSRD samples are subjected to large stresses, as is evidenced by McGrail *et al*.’s observation that RSRD cells are unable to recover from disruption through HU treatment and accumulate in S-phase 24.

The TRSS was therefore designed to minimize these non-physiological biases by excluding samples that might display exaggerated RS. The association of TRSS and SCS across tumor types, independent of proliferation and aneuploidies, suggests that the TRSS reliably captures tumorigenic RS that goes hand in hand with structural complexity. While the TRSS shows a slightly weaker association with the SCS compared to previous scores, it avoids key confounders and is more generalizable. Specifically, the repstress score is heavily influenced by proliferation, while the oncoRS signature was partially trained on oncogene amplification, which overlaps with the SCS definition. In contrast, the TRSS captures broader RS-related features, potentially at the cost of reduced signal in specific contexts.

Applying the TRSS to large datasets, we observed differences in proliferative cell cycle activity between primary tumors and cell lines associated with the TRSS. This finding is consistent with findings summarized in the introduction, where primary tumors generally exhibit increased proliferation and tumor growth, while cell lines demonstrate reduced cell viability and proliferation upon induction of RS and CIN. These results underscore the ability of the TRSS to capture proliferation-independent RS, despite being trained solely on cell line data and not explicitly optimized to account for context-specific proliferation effects.

Considering this additional evidence for a significant difference in RS-related proliferation between primary tumors and cell lines, a key question arises: Why does RS appear to impair long-term proliferation in cell lines but not in primary tumors? One key difference is the tumor microenvironment, which may be shaped to support the proliferation associated with RS and CIN by (i) reducing the function of T-cells 62, (ii) recruiting pro-tumor M2 macrophages 63,64, and (iii) signaling cancer-associated fibroblasts to provide energy-rich metabolites 65.

In TCGA, the TRSS was not associated with overall patient survival. A possible explanation for this could be the aneuploid adaptation mechanism associated with aggressive proliferation and hazardous chromosome arm alterations 60,61. This adaptation is also accompanied by a reduction in genomic instability, including RS, which may mask the impact of RS on patient survival. However, the TRSS identified a vulnerability to compounds inhibiting faithful DNA replication. The observed

correlations were consistent across multiple agents and are in line with the known role of RS in generating DNA lesions, which increase cellular dependence on intact DNA repair pathways 47. At the same time, agents introducing additional DSBs may overwhelm the DNA damage response. Given the interconnected nature of DNA repair pathways, a stronger response might be achieved by combinatorial treatments, which might also prevent adaptation to single target therapies 46.

Leveraging protein expressions, we found the *MSH2-6* protein complex to be one of the DNA repair components that is most strongly associated with the TRSS. Our findings suggest that this complex connects RS to an increase in NHEJ activity, potentially exacerbating CIN through low fidelity DNA damage repair. This is consistent with broader understanding that DSBs are generally resolved by two main pathways, HR and NHEJ 66. In context of HR deficient cells, which have been shown to also display reduced replication fork speeds 67,68, NHEJ likely becomes the compensatory repair mechanism for cell survival. Recent findings support this by showing that depleting *LIG4*, a central NHEJ factor, in an RS context resulted in an increase in apoptotic areas, while *BRCA2* inhibition only resulted in a minor effect 69.

Importantly, it has also been shown that *MSH6* seems to modulate NHEJ activity directly with experimental evidence demonstrating that inhibition of *MSH6* leads to decreased NHEJ activity 54. This relationship may be mediated via *MSH6*’s interaction with *Ku70* observed in both HeLa cells 54 and oocytes 70.

Extending this connection, Zhang et al. proposed that under RS conditions MutSα may block fork protecting factors causing DNA breaks and CIN upon misincorporations 71. Another study found that prostate cancers with increased MMR gene expression, including *MSH6*, displayed a higher incidence of deletions at different chromosome loci 72. Although the extent to which this RS-related NHEJ-activity is linked to CIN requires further investigation, these findings suggest that the TRSS will provide new insights into the role of RS in cancer.

## MATERIALS AND METHODS

### Development of the gene expression signature

1. Data quality and Processing of sequenced reads: We checked the RNA sequencing reads and removed any low-quality parts using FASTQC and Trimmomatic. We then quantified the gene expression counts using kallisto.

2. Normalization and batch correction: We shrinked variances toward a mean-variance trend, log2-normalized the counts, and corrected for batch effects of different study origins and cell lines.

3. Training and Testing Sets: We split the dataset into training and test data according to **Table 1** and Supplementary Table S2, while ensuring independent experiments by stratifying by treatment and cell line.

4. Identify most statistically RS-associated genes: We repeatedly (*n* times) selected a subset of *p*%. For each subset, we identified genes that changed in response to treatment. This differential gene expression was performed using the limma-voom pipeline 38,39. After these permutations we calculated the mean rank of the p-values of each gene.

5. Gene Selection: We selected the top *r* genes that were also assigned to RS-adjacent gene sets (Supplementary Table S3).

6. Train and validate model: Lastly, we used the selected genes to train a regularized logistic regression model with the elastic net coefficient *α*. We validated this approach using the unseen test dataset.

The hyperparameters (*n*, *p,* *r*, and *α*) as well as the choice of the mean rank for pre-selection were determined using 10-fold cross validation with the area under the receiver operating characteristic curve as performance metric.

### Calculation of RS scores

The repstress 15, the oncoRS 16 and the RS scores in this article were calculated as weighted linear combination of gene expression values. All gene expression data were first normalized using the variance-stabilizing transformation (vst) function from the DESeq2 package 73 in the R programming language. To control for technical variability, batch effects were removed using the removeBatchEffect function from the limma package 38. Each RS score was calculated by multiplying the transform, batch-corrected expression values by a set of predefined weights:

*score = w_1_ · x_1_ + w_2_ · x_2_ + ··· + w_n_ · x_n_*

where *x_1_, x_2_, …, x_n_* are the normalized expression values for the respective signature genes, and *w_1_, w_2_,…, w_n_* their corresponding weights.

• For oncoRS, all signature genes were weighted equally (*w_i_* = 1).

•For repstress, we used the gene-specific weights defined in (Supplementary Table S1) of the original publication.

•For the ARSS and TRSS, we used the gene weights listed in Tables 2 and 3, respectively.

### Statistical tests

Correlations were calculated using spearman correlation. Group differences were calculated using Wilcoxon rank-sum tests unless specified otherwise. Where applicable, p-values were adjusted for multiple testing using the Benjamini-Hochberg method.

### Gene Set Enrichment Analysis 

Differentially regulated pathways associated with gene signatures were determined using Gene Set Enrichment Analysis using the fgsea package on differentially expressed genes identified via the limma-voom pipeline 38,39. Single-sample GSEA was conducted using the gsva package to estimate gene set activity.

### Clinical analysis

To focus on intra-tumor effects, TRSS values were mean-centered within each tumor type to adjust for baseline differences. Samples in the first and fourth quartile were classified as low and high TRSS samples, respectively. The effect of RS status on survival was compared using a Kaplan-Meier analysis with the survival and survminer R packages.

For the analysis of the association of TRSS and drug response, area under the curve (AUC) values from the CTRP were min-max scaled to [0,1]. Drug sensitivity was then defined as 1-scaledAUC.

### Materials

The accession numbers for gene expressions used for generation and validation of RS signatures are listed in Table 1 & Supplementary Table S2 (accessed: 25. March 2024). Gene expression with RS samples with response defects are available at GSE59227.

Gene sets used were obtained from MsigDB 40,41: KEGG medicus gene set (MSigdb v2023.2) 56; Gene Ontology 44,45 and Hallmark (MSigdb v2023.1) 41

### Omics-data

Gene expression, DNA segments, mutational, proteomic, and clinical data in primary tumors generated by The Cancer Genome Atlas (TCGA) Research Network https://www.cancer.gov/tcga 74 were retrieved from the GDC data portal https://portal.gdc.cancer.gov/ (accessed: 03. July 2023).

Gene expression, and proteomic data in primary tumors generated by the National Cancer Institute Clinical Proteomic Tumor Analysis Consortium were retrieved from the PDC portal 75 https://proteomic.datacommons.cancer.gov/pdc/cptac-pancancer (accessed: 10. August 2024).

Gene expression data in cell lines compiled in the Cancer cell line encyclopedia 76 was retrieved through the DepMap data portal https://depmap.org/portal (accessed: 23. August 2023).

### TCGA derivative data:

SCS: Distinct and Common Features of Numerical and Structural across Different Cancer Types 18.

Proliferation rate, HRD score, aneuploidy score: The immune landscape of cancer 77 (accessed: 01. July 2024).

Amplification and Deletion calls: Oncogenic Signaling Pathways in The Cancer Genome Atlas 78 (accessed: 27. June 2024).

### CCLE derivative data:

We retrieved cell line drug sensitivities from the Cancer Therapeutics Response Portal (CTRP) version 2 through the National Cancer Institute’s data portal https://ctd2-data.nci.nih.gov/ Public/Broad/CTRPv2.0_2015_ctd2_ExpandedDataset/ (accessed: 23. August 2023).

## CONFLICT OF INTEREST

The authors declare no competing interests.

## SUPPLEMENTAL MATERIAL 

Click here for supplemental data file.

Click here for supplemental data file.

All supplemental data for this article are available online at https://www.cell-stress.com/researcharticles/2025a-jungk-cell-stress/
