# Supplementary material for: Quantifying replication stress in cancer without proliferation confounding
Source: Cell Stress. 2025 Oct 28;9:201–15. doi: 10.15698/cst2025.10.312 (PMC12632277; doi:10.15698/cst2025.10.312)
Supplement: Supplementary file 1 [file ces-09-201-s01.pdf]

Supplementary Information

Supplementary Methods

Assessments of effect of hallmark activity on RS score-SCS association

To evaluate the robustness of the association between TRSS or repstress score and the SCS to the activity of hallmark gene pathways. We constructed a series of linear regression models. The baseline model has the following form:

$$SCS_i = \beta_0 \cdot Tumor\_Type_i + \beta_1 \cdot RS_i$$

Additional models were then fit including a term for the activity of hallmark gene sets:

$$SCS_i = \beta_0 \cdot Tumor\_Type_i + \beta_1 \cdot RS_i + \beta_2 \cdot HM\_activity_i$$

For each model the coefficient of the RS score was normalized relative to the baseline model (default coefficient set to 1). 95% confidence intervals were added to visualize the uncertainty of the coefficient.

Supplementary Results

Replication stress-related genomic alterations

We examined genomic alterations associated with the Tumorigenic RS score (TRSS), to identify potential genomic causes and consequences RS. Mutations that are associated with the TRSS include the deubiquitinase *USP13* (Supplementary Figure S5A), likely compromising genomic stability by reducing its ability to stabilize cell cycle regulating proteins (e.g. *SKP2*, *AURKB*) and DNA damage response genes (e.g. *RAP80*, *AURKB*, *TP53*) [80]. Similarly, mutations in *DDX19A* might impair the cell's ability to resolve R-loops – RNA-DNA hybrid structures – promoting DSBs and increasing chromosomal instability [81]. Mutations in *CEP250* (*C-NAP1*) may disrupt centrosome cohesion, especially in cells with RS and DNA damage, which have both been shown to induce premature centriole disengagement [3, 26, 82].

Similarly, we also see gene losses associated with the TRSS (Supplementary Figure S5B). One of the genes with deletions as classified by the oncokb database [78], is *MYO6*, which protects replication forks upon RS to maintain genomic stability [83]. A loss of *MYO6* could therefore lead to induction of genomic instability and intra-tumor heterogeneity. Deletion of *CYB5R4* sensitizes cells to camptothecin, an inducer of RS that leads to inhibited DNA unwinding and torsional stress [9]. This suggests a role for *CYB5R4* in regulating the DNA damage response and maintaining replication fork progression [84].

Amplifications of genes associated with the TRSS included *SOX4* and PRL (Supplementary Figure S5C). *SOX4* overexpression plays a dual role in the *TP53* pathway: it stabilizes *TP53* by blocking *MDM2*-mediated ubiquitination, while simultaneously inhibiting *TP53*'s transcriptional activation of pro-apoptotic targets like *BAX* [85, 86]. This deregulation of the *TP53* pathway may prevent the faithful induction of apoptosis in DNA-damaged cells, thereby promoting genomic instability. Similarly, prolactin (*PRL*) overexpression in breast cancers may promote cancer cell survival and proliferation in the context of *TP53* loss [87, 88].

These gene alterations highlight how loss of function through mutations and deletions may impair fork stability and DNA damage checkpoint activity, while amplifications of

survival-promoting factors allow cells to evade apoptosis, leading to increased genomic instability and tumorigenesis.

Supplementary Figures

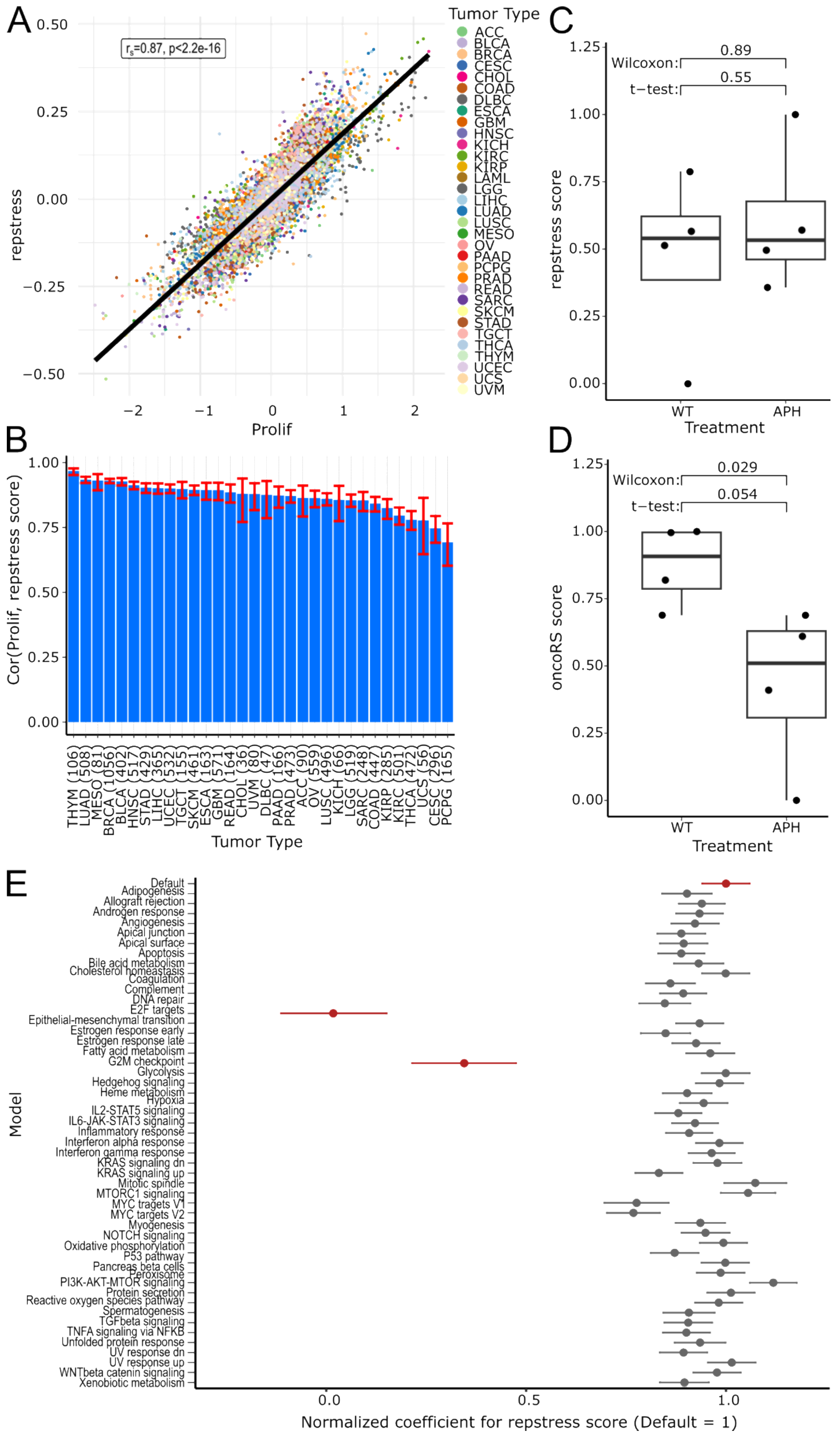

Supplementary Figure S1: Repstress score is mainly driven by proliferation and cell cycle activity.

(A) Scatterplot of the repstress score versus a measure of proliferation across TCGA tumor types. Scores were mean-centered within each tumor type. **(B)** Spearman correlation between the repstress score and the proliferative score (y-axis) in different TCGA tumor types (x-axis). **(C, D)** Comparison of repstress score **(C)** and oncoRS score **(D)** and between aphidicolin-treated (200 nM; APH) and untreated (WT) samples. **(E)** Regression coefficient for the association between repstress score and SCS. The default model includes only the tumor types as a covariate, while all other models include the activity in one hallmark gene set. Coefficients for the score are expressed relative to the default model (set to 1). Errorbars indicate the 95% confidence intervals.

TCGA: The Cancer Genome Atlas; SCS: Structural Complexity Score

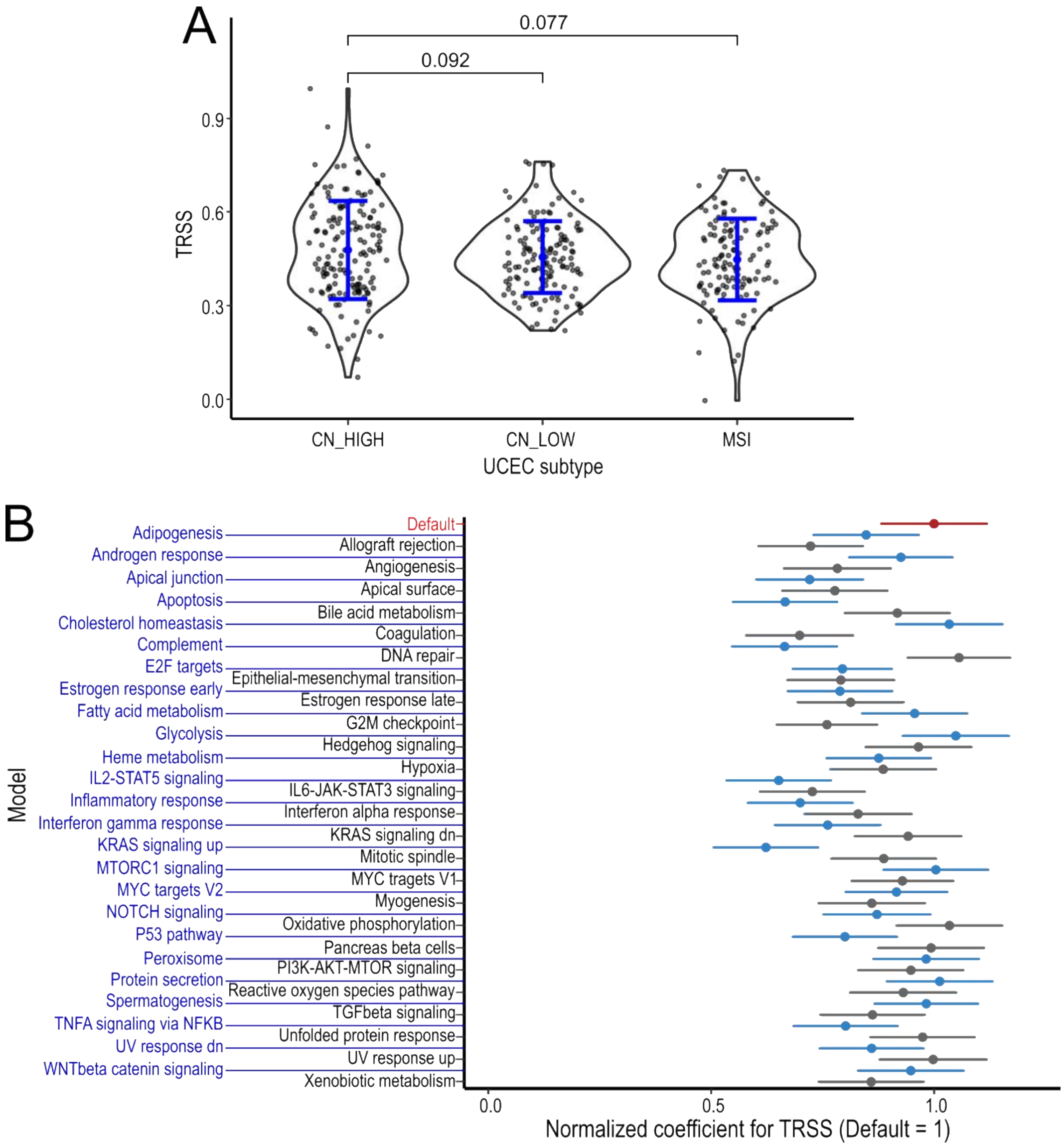

Supplementary Figure S2: TRSS in genomic instability-related subtypes and hallmark confounders of TRSS-SCS association.

(A) Comparison of TRSS distribution in MSI, and CN low subtypes with the CN high subtype in UCEC cancers of the TCGA dataset. **(B)** Regression coefficient for the association between TRSS and SCS. The default model includes only the tumor types as a covariate, while all other models include the activity in one hallmark gene set. Coefficients for the score are expressed relative to the default model (set to 1). Error bars indicate the 95% confidence intervals.

TRSS: Tumorigenic replication stress signature; SCS: Structural Complexity Score; MSI: Microsatellite instability; CN: Copy number; UCEC: Uterine Corpus Endometrial Carcinoma; TCGA: The Cancer Genome Atlas

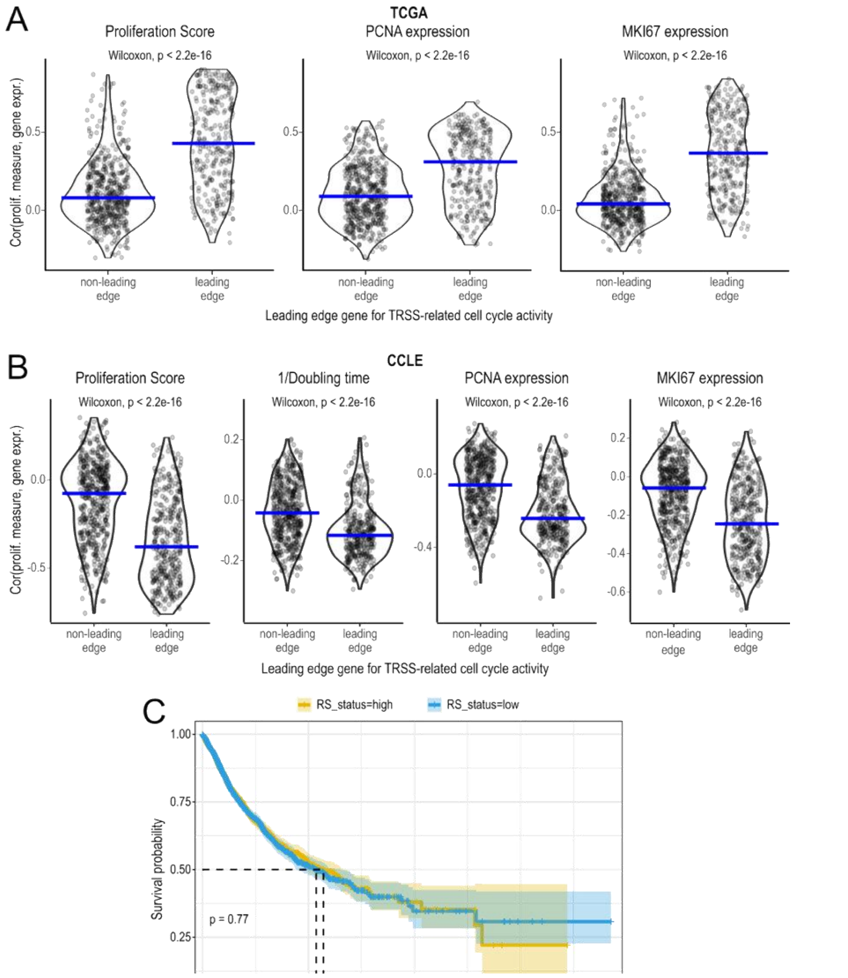

Supplementary Figure S3: Association of cell cycle genes to proliferation measures in primary tumors and cell lines.

Each datapoint describes the correlation of a cell cycle gene to a proliferation score (left), PCNA marker expression (center) and MKI67 marker expression (right). This correlation is compared between leading-edge genes (TRUE) and non-leading-edge genes (FALSE) in an enrichment analysis for the tumorigenic replication stress signature in primary tumors from TCGA **(A)** and cell lines from the CCLE **(B)**, where the inverse of the doubling time is added as proliferative measure. **(C)** Kaplan-Meier survival plot comparing patients in the highest and lowest quartile of TRSS. TCGA: The Cancer Genome Atlas; CCLE: Cancer cell line encyclopedia; TRSS: Tumorigenic replication stress signature

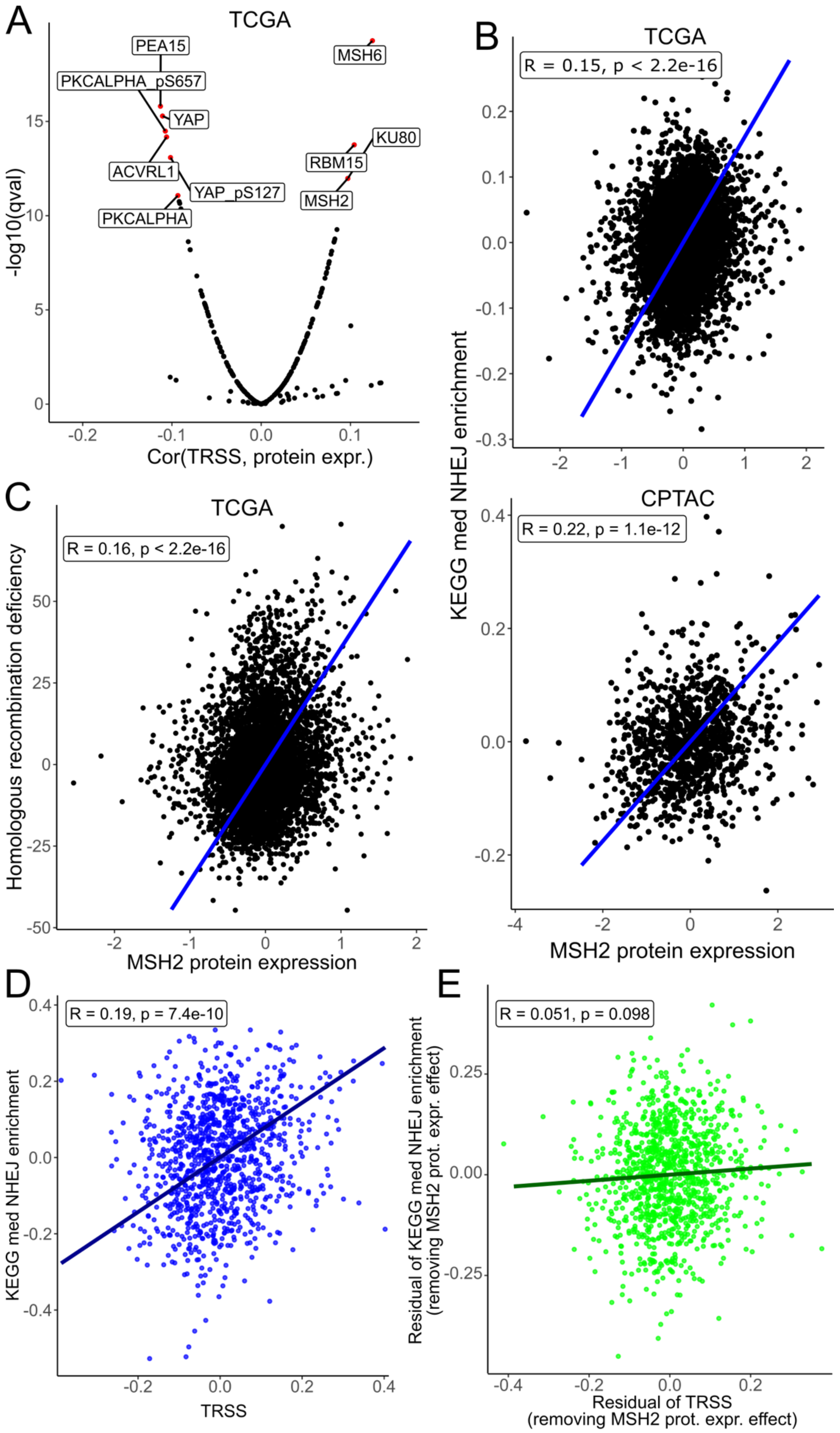

Supplementary Figure S4: The TRSS is associated with MSH2 protein which correlations with NHEJ activity and homologous recombination deficiency.

(A) Volcano plot visualizing the spearman correlation and q-values of TCGA protein expressions to the TRSS. **(B)** Activity in the KEGG medicus NHEJ gene set compared to the

protein expression of MSH2 in TCGA (top) and CPTAC (bottom). **(C)** Amount of genomic scars indicating a deficiency of homologous recombination repair compared to the protein expression of MSH2. **(D)** Activity in the KEGG medicus NHEJ gene set compared to the TRSS in CPTAC. **(E)** Activity in the KEGG medicus NHEJ gene set compared to the TRSS after adjusting for the effects of MSH2 expression in CPTAC.

TRSS: Tumorigenic replication stress signature; TCGA: The Cancer Genome Atlas; CPTAC: Clinical Proteomic Tumor Analysis Consortium; NHEJ: Non-homologous end-joining; KEGG: Kyoto Encyclopedia of Genes and Genomes

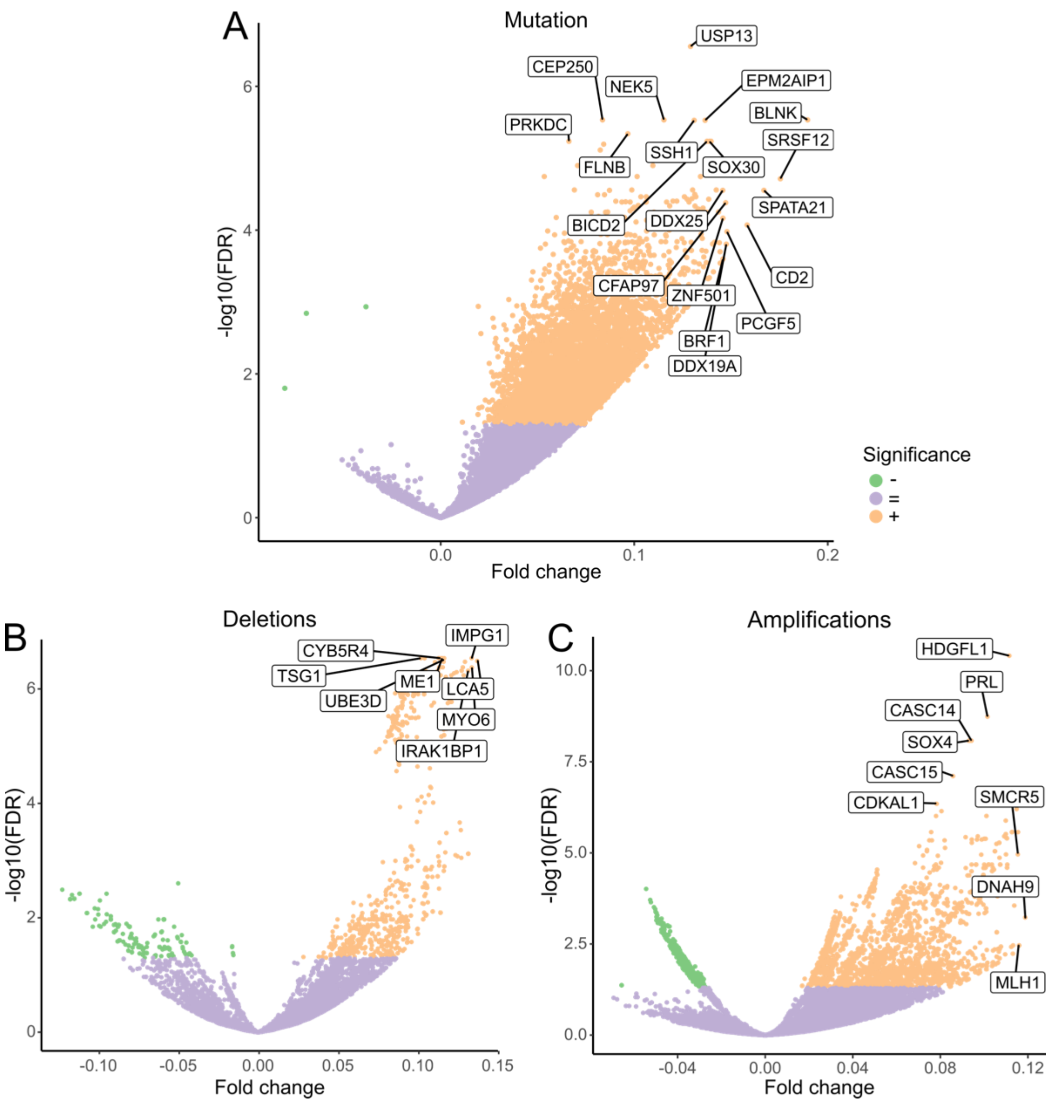

Supplementary Figure S5: Genomic alterations associated with the tumorigenic RS score (TRSS).

Displayed is the association of each gene's alteration to the TRSS in TCGA. The x-axis represents the fold change of mutations **(A)**, deletions **(B)** and amplifications **(C)** in association with the TRSS. The y-axis represents the –log10 transformed, multiple testing adjusted p-value.

TRSS: Tumorigenic replication stress signature; TCGA: The Cancer Genome Atlas

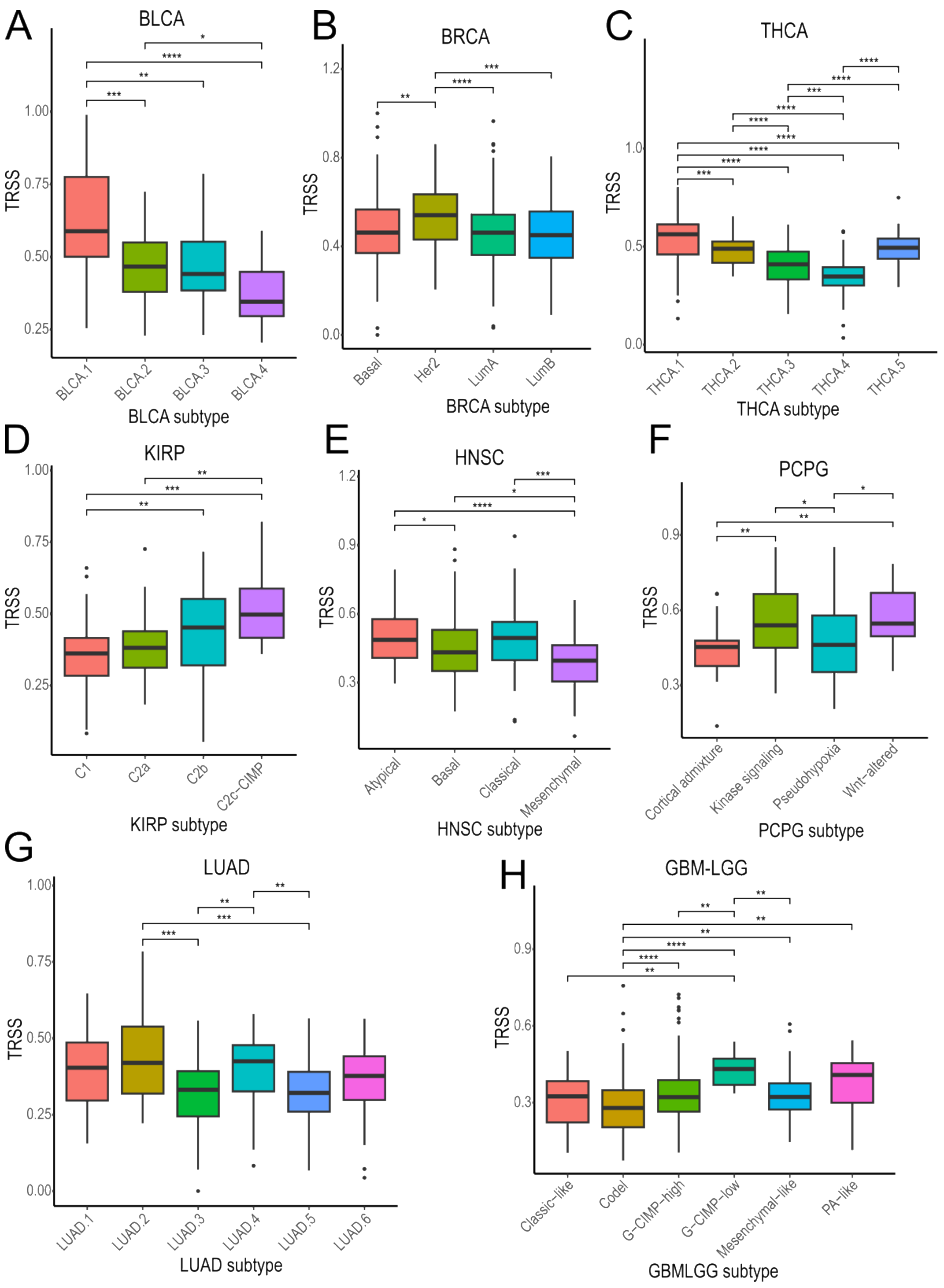

Supplementary Figure S6: Differences in TRSS levels between molecular subtypes in various TCGA tumor types.

The boxplots display the TRSS distributions across subtypes in bladder urothelial carcinoma **(A)**, breast invasive carcinoma **(B)**, thyroid carcinoma **(C)**, kidney renal clear cell carcinoma **(D)**, head and neck squamous cell carcinoma **(E)**, Pheochromocytoma and paraganglioma **(F)**, lung adenocarcinoma **(G)**, and glioblastoma multiforme and brain lower grade glioma **(H)**. The subtypes were gathered from the PanCancerAtlas_subtypes function of the TCGAbiolinks package in R. Significance levels were calculated using a Wilcoxon

rank test and indicated by stars as follows: **** $p \leq 0.0001$; *** $p \leq 0.001$; ** $p \leq 0.01$; * $p \leq 0.05$

TRSS: Tumorigenic replication stress signature; TCGA: The Cancer Genome Atlas

Supplementary Tables

Supplementary Table S1: Spearman correlation of repstress signature genes to three proliferation measures.

gene	cor prolif	pval prolif	cor *PCNA*	pval *PCNA*	cor *MKI67*	pval *MKI67*	cell cycle gene
CCNA2	0.89	0.00e+00	0.53	0.00e+00	0.74	0.00e+00	TRUE
GINS1	0.79	0.00e+00	0.69	0.00e+00	0.58	0.00e+00	TRUE
AURKB	0.78	0.00e+00	0.55	0.00e+00	0.65	0.00e+00	TRUE
ORC6	0.74	0.00e+00	0.51	0.00e+00	0.51	0.00e+00	FALSE
RRM1	0.53	0.00e+00	0.43	0.00e+00	0.47	0.00e+00	TRUE
RFC5	0.55	0.00e+00	0.48	0.00e+00	0.38	2.30e-280	FALSE
POLA1	0.44	0.00e+00	0.33	5.06e-208	0.43	0.00e+00	TRUE
SUV39H1	0.4	1.08e-318	0.4	7.35e-319	0.31	4.93e-187	TRUE
RMI1	0.42	0.00e+00	0.31	1.26e-190	0.33	2.80e-210	TRUE
SRSF1	0.41	0.00e+00	0.27	9.25e-139	0.28	2.79e-153	FALSE
MTF2	0.3	2.70e-173	0.12	1.31e-27	0.18	2.21e-59	FALSE
POLD4	0.21	3.48e-86	0.09	1.91e-16	0.18	1.33e-61	FALSE
PRPS1	0.16	2.02e-49	0.18	2.31e-64	0.09	7.24e-17	FALSE
LIG3	0.16	7.88e-47	0.04	8.50e-05	0.15	1.36e-44	TRUE
TNPO2	0.06	2.82e-09	-0.04	1.93e-04	0.15	6.59e-41	FALSE
POLE4	-0.05	5.16e-06	-0.19	2.86e-70	0.09	1.68e-16	FALSE
GADD45G	-0.22	2.54e-93	-0.07	3.31e-10	-0.21	1.14e-81	TRUE

A gene was labelled as cell cycle gene if it appeared in Gene Ontology gene sets containing the phrase “cell cycle”.

Supplementary Table S2: Samples with replication stress-induction by gene amplification or knockout, or treatment with replication stress-inducing drugs for generating a replication stress signature.

Study id	cell line	RS technique	drug dosage	GEO series
GSE150354	BJ	APH	200 nM	GSE150354
GSE150639	hESC	HU	150 μM	GSE150639
GSE159826	RPE_1	APH	200 nM	GSE159826
GSE160277	EBV_lymph	APH	300 nM	GSE160277
GSE168689	BJ	APH	400 nM	GSE168689
GSE168689	RPE_1	APH	400 nM	GSE168689
GSE171845	RPE_1	CCNE1		GSE171845
GSE182775	U2OS	HU	2 mM	GSE182775
GSE185512	RPE_1	CDC25A, CCNE1, MYC		GSE185512

**GSE185512**	RPE_1_NOp53	CDC25A, CCNE1, MYC	GSE185512
**GSE185512**	BT549	CDC25A, CCNE1, MYC	GSE185512
**GSE185512**	MDA_MB231	CDC25A, CCNE1, MYC	GSE185512
**GSE185512**	HCC1806	CDC25A, CCNE1, MYC	GSE185512
**GSE244103**	H446	DHX9_KO	GSE244103
**GSE244103**	H82	DHX9_KO	GSE244103
**GSE244103**	H196	DHX9_KO	GSE244103
**GSE252544**	Mia_PACA_2	CDA_KO	GSE252544
**GSE292334**	HCT116	SMAD4_KO	GSE292334
**GSE292334**	HT29	SMAD4_KO	GSE292334

Supplementary Table S3: Gene sets used as prior knowledge included in the training of the replication stress models.

**Gene_set**	**size**
GOBP_DNA_REPLICATION_CHECKPOINT_SIGNALING	18
GOBP_DNA_SYNTHESIS_INVOLVED_IN_DNA_REPAIR	40
GOBP_DNA_REPLICATION	279
GOBP_DNA_TEMPLATED_DNA_REPLICATION	161
GOBP_MITOCHONDRIAL_DNA_REPLICATION	14
GOBP_DNA_UNWINDING_INVOLVED_IN_DNA_REPLICATION	21
GOBP_DNA_REPLICATION_SYNTHESIS_OF_RNA_PRIMER	7
GOBP_DNA_REPLICATION_INITIATION	37
GOBP_DNA_STRAND_ELONGATION_INVOLVED_IN_DNA_REPLICATION	15
GOBP_REGULATION_OF_DNA_REPLICATION	136
GOBP_DNA_REPAIR	584
GOBP_REGULATION_OF_DNA_REPAIR	214
GOBP_DNA_DEALKYLATION_INVOLVED_IN_DNA_REPAIR	9
GOBP_DNA_REPLICATION_DEPENDENT_CHROMATIN_ASSEMBLY	6
GOBP_NEGATIVE_REGULATION_OF_DNA_REPLICATION	32
GOBP_POSITIVE_REGULATION_OF_NUCLEAR_CELL_CYCLE_DNA_REPLICATION	6
GOBP_REGULATION_OF_DNA_TEMPLATED_DNA_REPLICATION_INITIATION	15
GOBP_REPLICATION_FORK_PROCESSING	46
GOBP_REGULATION_OF_NUCLEAR_CELL_CYCLE_DNA_REPLICATION	13
GOBP_MITOTIC_DNA_REPLICATION_CHECKPOINT_SIGNALING	10
GOBP_MITOCHONDRIAL_DNA_REPAIR	5
GOBP_CELL_CYCLE_DNA_REPLICATION	44
GOBP_DNA_TEMPLATED_DNA_REPLICATION_MAINTENANCE_OF_FIDELITY	56
GOBP_NEGATIVE_REGULATION_OF_DNA_REPAIR	36
GOBP_POSITIVE_REGULATION_OF_DNA_REPAIR	130
GOBP_POSITIVE_REGULATION_OF_DNA_REPLICATION	39
GOBP_REPLICATION_FORK_PROTECTION	10
GOBP_DNA_LIGATION_INVOLVED_IN_DNA_REPAIR	5

GOBP_R_LOOP_PROCESSING	6
GOBP_REGULATION_OF_DNA_TEMPLATED_DNA_REPLICATION	58
GOBP_DNA_STRAND_RESECTION_INVOLVED_IN_REPLICATION_FORK_PROCESSING	8
GOBP_DNA_REPAIR_DEPENDENT_CHROMATIN_REMODELING	6
GOBP_CELL_CYCLE_DNA_REPLICATION_INITIATION	5
GOBP_MITOTIC_DNA_REPLICATION	14
GOBP_NEGATIVE_REGULATION_OF_DNA_TEMPLATED_DNA_REPLICATION	16
GOBP_POSITIVE_REGULATION_OF_DNA_TEMPLATED_DNA_REPLICATION	13
GOCC_ORIGIN_RECOGNITION_COMPLEX	9
GOCC_REPLICATION_FORK	66
GOCC_DNA_REPLICATION_FACTOR_A_COMPLEX	5
GOCC_DNA_REPLICATION_FACTOR_C_COMPLEX	5
GOCC_DNA_REPLICATION_PREINITIATION_COMPLEX	12
GOCC_NUCLEAR_REPLICATION_FORK	35
GOCC_DNA_REPAIR_COMPLEX	22
GOMF_DNA_REPLICATION_ORIGIN_BINDING	16
HP_DEFECTIVE_DNA_REPAIR_AFTER_ULTRAVIOLET_RADIATION_DAMAGE	14
HP_ABNORMALITY_OF_DNA_REPAIR	18
KAUFFMANN_DNA_REPAIR_GENES	230
KAUFFMANN_DNA_REPLICATION_GENES	146
MATZUK_MEIOTIC_AND_DNA_REPAIR	39
WHITFIELD_CELL_CYCLE_M_G1	126
KEGG_DNA_REPLICATION	36
KEGG_HOMOLOGOUS_RECOMBINATION	28
KEGG_PYRIMIDINE_METABOLISM	98
KEGG_MEDICUS_REFERENCE_DNA_REPLICATION_LICENSING	24
KEGG_MEDICUS_REFERENCE_DNA_REPLICATION_TERMINATION	21
KEGG_MEDICUS_REFERENCE_ORIGIN_UNWINDING_AND_ELONGATION	30
REACTOME_ACTIVATION_OF_ATR_IN_RESPONSE_TO_REPLICATION_STRESS	37
REACTOME_ASSEMBLY_OF_THE_ORC_COMPLEX_AT_THE_ORIGIN_OF_REPLICATION	68
REACTOME_CDC6_ASSOCIATION_WITH_THE_ORC_ORIGIN_COMPLEX	8
REACTOME_CELL_CYCLE	692
REACTOME_CHK1_CHK2_CDS1_MEDIATED_INACTIVATION_OF_CYCLIN_B_CDK1_COMPLEX	13
REACTOME_CONVERSION_FROM_APC_C_CDC20_TO_APC_C_CDH1_IN_LATE_ANAPHASE	20
REACTOME_CYCLIN_A_B1_B2_ASSOCIATED_EVENTS_DURING_G2_M_TRANSITION	25
REACTOME_DISEASES_OF_DNA_REPAIR	51
REACTOME_DISEASES_OF_MISMATCH_REPAIR_MMR	5
REACTOME_DNA_DAMAGE_RECOGNITION_IN_GG_NER	38
REACTOME_DNA_REPAIR	332
REACTOME_DNA_REPLICATION	187
REACTOME_DNA_REPLICATION_INITIATION	8
REACTOME_DNA_REPLICATION_PRE_INITIATION	159
REACTOME_E2F_ENABLED_INHIBITION_OF_PRE_REPLICATION_COMPLEX_FORMATION	9
REACTOME_E2F_MEDIATED_REGULATION_OF_DNA_REPLICATION	22
REACTOME_FANCONI_ANEMIA_PATHWAY	39
REACTOME_FORMATION_OF_SENESCENCE_ASSOCIATED_HETEROCHROMATIN_FOCI_SAHF	17
REACTOME_FORMATION_OF_TC_NER_PRE_INCISION_COMPLEX	53
REACTOME_G1_S_DNA_DAMAGE_CHECKPOINTS	68
REACTOME_G2_M_DNA_DAMAGE_CHECKPOINT	94
REACTOME_G2_M_DNA_REPLICATION_CHECKPOINT	5
REACTOME_GAP_FILLING_DNA_REPAIR_SYNTHESIS_AND_LIGATION_IN_GG_NER	25
REACTOME_HDR_THROUGH_SINGLE_STRAND_ANNEALING_SSA	37

REACTOME_HOMOLOGOUS_DNA_PAIRING_AND_STRAND_EXCHANGE	43
REACTOME_MITOTIC_SPINDLE_CHECKPOINT	111
REACTOME_RECOGNITION_OF_DNA_DAMAGE_BY_PCNA_CONTAINING_REPLICATION_COMPLEX	30
REACTOME_SUMOYLATION_OF_DNA_REPLICATION_PROTEINS	46
REACTOME_TP53_REGULATES_TRANSCRIPTION_OF_DNA_REPAIR_GENES	62
WP_DNA_REPAIR_PATHWAYS_FULL_NETWORK	120
WP_DNA_REPLICATION	42
HALLMARK_DNA_REPAIR	150

Supplementary Table S4: Tumorigenic Replication Stress Signature associated enrichment of cell cycle-related gene sets in the gene ontology biological process (GOBP) and hallmark collections in primary tumors (TCGA) and cell lines (CCLE).

Pathway	padj	NES	dataset
HALLMARK E2F targets	2.88E-04	-1.73	CCLE
HALLMARK MYC targets V1	5.23E-03	-1.58	CCLE
GOBP negative regulation of mitotic cell cycle phase transition	0.01	-1.57	CCLE
HALLMARK G2M checkpoint	0.02	-1.50	CCLE
GOBP negative regulation of cell cycle process	0.06	-1.38	CCLE
GOBP mitotic cell cycle checkpoint signaling	0.24	-1.30	CCLE
GOBP negative regulation of mitotic cell cycle	0.18	-1.30	CCLE
GOBP regulation of mitotic cell cycle phase transition	0.16	-1.27	CCLE
GOBP mitotic cell cycle phase transition	0.27	-1.19	CCLE
GOBP cell cycle checkpoint signaling	0.44	-1.18	CCLE
GOBP cell cycle G2M phase transition	0.49	-1.17	CCLE
GOBP regulation of cell cycle G2M phase transition	0.51	-1.15	CCLE
HALLMARK MYC targets V2	0.51	-1.14	CCLE
GOBP negative regulation of cell cycle	0.51	-1.10	CCLE
GOBP regulation of cell cycle phase transition	0.51	-1.09	CCLE
GOBP meiotic cell cycle process	0.54	-1.08	CCLE
GOBP cell cycle phase transition	0.51	-1.07	CCLE
GOBP meiotic cell cycle	0.59	-1.06	CCLE
GOBP negative regulation of cell cycle G2M phase transition	0.63	-1.05	CCLE
GOBP cell cycle DNA replication	0.63	-1.04	CCLE
GOBP regulation of mitotic cell cycle	0.96	-0.94	CCLE
GOBP positive regulation of cell cycle process	0.99	-0.82	CCLE
GOBP regulation of mitotic cell cycle spindle assembly checkpoint	0.98	-0.79	CCLE
GOBP positive regulation of cell cycle phase transition	0.99	-0.65	CCLE
GOBP chromosome organization involved in meiotic cell cycle	0.99	-0.64	CCLE
GOBP meiosis I cell cycle process	0.99	0.80	CCLE
GOBP regulation of cell cycle checkpoint	0.98	0.81	CCLE
GOBP positive regulation of cell cycle G2M phase transition	0.98	0.82	CCLE
GOBP positive regulation of cell cycle	0.99	0.84	CCLE
HALLMARK MYC targets V1	0.40	1.03	TCGA
GOBP positive regulation of cell cycle checkpoint	0.62	1.07	CCLE
GOBP negative regulation of cell cycle	9.45E-04	1.40	TCGA
GOBP regulation of mitotic cell cycle	6.49E-05	1.41	TCGA
HALLMARK MYC targets V2	0.05	1.42	TCGA
GOBP positive regulation of cell cycle phase transition	0.01	1.45	TCGA

GOBP regulation of mitotic cell cycle phase transition	2.38E-05	1.55	TCGA
GOBP negative regulation of mitotic cell cycle	2.17E-04	1.56	TCGA
GOBP negative regulation of mitotic cell cycle phase transition	4.83E-04	1.57	TCGA
GOBP mitotic cell cycle phase transition	9.43E-08	1.64	TCGA
GOBP positive regulation of cell cycle	2.80E-06	1.64	TCGA
GOBP negative regulation of cell cycle G2M phase transition	5.88E-03	1.69	TCGA
GOBP positive regulation of cell cycle G2M phase transition	0.02	1.70	TCGA
GOBP regulation of cell cycle phase transition	2.96E-08	1.70	TCGA
GOBP positive regulation of cell cycle process	1.44E-05	1.72	TCGA
GOBP cell cycle phase transition	2.67E-10	1.72	TCGA
GOBP negative regulation of cell cycle process	2.20E-06	1.72	TCGA
GOBP regulation of mitotic cell cycle spindle assembly checkpoint	0.01	1.82	TCGA
GOBP regulation of cell cycle G2M phase transition	1.52E-04	1.84	TCGA
GOBP cell cycle G2M phase transition	2.83E-05	1.84	TCGA
GOBP positive regulation of cell cycle checkpoint	7.79E-03	1.88	TCGA
GOBP regulation of cell cycle checkpoint	1.66E-04	2.08	TCGA
GOBP mitotic cell cycle checkpoint signaling	3.00E-07	2.09	TCGA
GOBP cell cycle checkpoint signaling	7.94E-09	2.15	TCGA
GOBP meiotic cell cycle	2.65E-12	2.38	TCGA
GOBP meiotic cell cycle process	1.40E-10	2.39	TCGA
GOBP cell cycle DNA replication	3.92E-06	2.40	TCGA
GOBP chromosome organization involved in meiotic cell cycle	3.00E-07	2.48	TCGA
GOBP meiosis I cell cycle process	1.48E-09	2.56	TCGA
HALLMARK G2M checkpoint	8.93E-25	3.01	TCGA
HALLMARK E2F targets	8.36E-26	3.05	TCGA

Supplementary Table S5: Association of mismatch repair protein expression to the Tumorigenic Replication Stress Signature.

Protein	cor	pval	qval
MSH6	0.302	6.42E-24	3.33E-20
MSH2	0.282	5.30E-21	1.20E-17
MLH1	0.147	3.48E-06	5.37E-05
PMS1	0.116	4.08E-04	3.16E-03
MSH3	0.092	2.58E-03	1.46E-02
PMS2	0.088	6.15E-03	2.95E-02
MLH3	-0.092	1.70E-01	3.56E-01
